# Multi-Targeting Approach in Selection of Potential Molecule for COVID-19 Treatment

**DOI:** 10.3390/v15010213

**Published:** 2023-01-12

**Authors:** Varalakshmi Velagacherla, Akhil Suresh, Chetan Hasmukh Mehta, Usha Y. Nayak, Yogendra Nayak

**Affiliations:** 1 Department of Pharmaceutics, Manipal College of Pharmaceutical Sciences, Manipal Academy of Higher Education, Udupi 576104, India; 2 Manipal Centre for Infectious Diseases, Prasanna School of Public Health, Manipal Academy of Higher Education, Udupi 576104, India; 3 Department of Pharmacology, Manipal College of Pharmaceutical Sciences, Manipal Academy of Higher Education, Udupi 576104, India

**Keywords:** SARS-CoV-2, molecular docking, prime MM-GBSA, induced-fit docking, molecular dynamics simulation

## Abstract

The coronavirus disease (COVID-19) is a pandemic that started in the City of Wuhan, Hubei Province, China, caused by the spread of coronavirus (SARS-CoV-2). Drug discovery teams around the globe are in a race to develop a medicine for its management. It takes time for a novel molecule to enter the market, and the ideal way is to exploit the already approved drugs and repurpose them therapeutically. We have attempted to screen selected molecules with an affinity towards multiple protein targets in COVID-19 using the Schrödinger suit for in silico predictions. The proteins selected were angiotensin-converting enzyme-2 (ACE2), main protease (M^Pro^), and spike protein. The molecular docking, prime MM-GBSA, induced-fit docking (IFD), and molecular dynamics (MD) simulations were used to identify the most suitable molecule that forms a stable interaction with the selected viral proteins. The ligand-binding stability for the proteins PDB-IDs 1ZV8 (spike protein), 5R82 (M^pro^), and 6M1D (ACE2), was in the order of nintedanib > quercetin, nintedanib > darunavir, nintedanib > baricitinib, respectively. The MM-GBSA, IFD, and MD simulation studies imply that the drug nintedanib has the highest binding stability among the shortlisted. Nintedanib, primarily used for idiopathic pulmonary fibrosis, can be considered for repurposing for us against COVID-19.

## 1. Introduction

The novel coronavirus disease (COVID-19) was first identified in Wuhan city of China. The first case was reported in December 2019. A woman aged 54, diagnosed with severe acute respiratory distress syndrome with asthenia, fever, and other respiratory difficulties, such as a low respiratory rate and a decreased oxygen saturation in the body, was found to be infected with the virus. This virus was first named the ‘novel coronavirus’ (nCoV); later, the nomenclature was changed to ‘severe acute respiratory syndrome corona virus-2’ (SARS-CoV-2). The World Health Organization declared this pandemic disease due to SARS-CoV-2 as COVID-19 [1]. Severe Acute Respiratory Syndrome (SARS), Middle Eastern Respiratory Syndrome (MERS), and COVID-19 are zoonotic and have bats as a common origin. All three viral diseases share common symptoms; they are diagnosed by taking nasal or throat swabs and then using RT-PCR to determine the presence of viral genetic material [2]. The person-to-person transmission of SARS-CoV-2 occurs via nasal droplets and by direct contact with an infected patient or the materials and surfaces used and touched directly by the infected person. The major symptoms seen in COVID-19 patients are fever, cough, fatigue, headache, cardiac injury, hypoxemia, lymphopenia, dyspnoea, diarrhoea, rhinorrhea, pneumonia, respiratory distress syndrome, sore throat, sneezing, and RNAaemia [3]. SARS-CoV-2 gains access to the body via the nose, mouth, or eyes, and from there, it moves into alveolar sacs and further into the alveolar cells of the lungs. The SARS-CoV-2 uses its spike glycoprotein to enter into cells. The spike protein (S-protein) of the coronavirus is composed of transmembrane glycoprotein, which is trimetric in nature and protrudes from the surface of the virus. The viral S-protein has a high affinity to ACE2 receptors, mainly in type-II alveolar cells. The virus is then internalized by membrane fusion or endocytosis [4]. The main protease (M^pro^) is a viral protease that activates a series of events with RNA-dependent RNA polymerase (RdRp), which helps in the replication of the viral genetic material and produces multiple virus copies [5]. Hence, S-protein, ACE2, M^pro^, and RdRp are important druggable targets in drug discovery for COVID-19. 

SARS-CoV-2 proteins are of two classes, structural and non-structural proteins (nsp). The non-structural proteins include M^pro^, papain-like protease (PLpro), nsp13 (also known as helicase), nsp12 (also known as RdRp), nsp14 (also known as N-terminal exoribonuclease and C-terminal guanine-N7 methyltransferase), nsp15 (also known as uridylate-specific endoribonuclease), nsp16 (also known as 2’-O-methyltransferase), and nsp10, while the structural proteins include S-protein, envelope small membrane protein (E), membrane protein (M), and nucleocapsid protein (N) [6]. Clinical trials are ongoing to identify a suitable drug molecule that can combat the problems associated with COVID-19 [7]. Though the repurposing of drugs such as remdesivir, favipiravir, molnupiravir, nirmatrelvir–ritonavir, and dexamethasone has been approved for the treatment of COVID-19, they have major limitations. Remdesivir has adverse reactions, such as liver and kidney toxicity [8]. Favipiravir provides better rates of viral clearance; however, its liver and kidney toxicities were similar to remdesivir [9]. Both remdesivir and favipiravir are expensive in terms of cost-effectiveness. The newer drugs, such as molnupiravir or the nirmatrelvir–ritonavir combination, have been found to have similar adverse drug reactions and are not tolerated in all patients [10]. Dexamethasone has its own limitations, such as the fact that it cannot be used in immunocompromised patients [11]. There is no complete cure for COVID-19 with the available drugs, but these drugs reduce hospitalization and the death rate [12]. As per the studies, oral nirmatrelvir plus ritonavir and intravenous remdesivir is reported to be the better choice, followed by molnupiravir. However, most of these drugs just target the early steps of the life cycle or the early phase of infection, and there are no excellent drugs that provide a long-lasting suppression of viral replication [13,14].

Computational modeling helps to identify a potentially useful molecule from a large library of molecules in less time by using various tools, such as molecular docking, Prime molecular mechanics generalized Born surface area (Prime MM-GBSA), induced-fit docking (IFD), followed by molecular dynamic (MD) simulation for the selected molecules [15]. In our earlier attempts, we found that penicillin had the potential to bind to M^pro^ consistently, while phenoxymethylpenicillin and carbenicillin had high potential to act as anti-COVID-19 agents, which can be used alongside antiviral agents [16]. In another study, we studied the in silico repurposing of USFDA-approved drugs such as aprepitant, barnidipine, tipiracil, arbutin, and terbutaline by determining their binding ability to M^pro^, thereby acting as anti-COVID-19 agents [17]. We have also attempted to predict drugs that target RdRp, and found pitavastatin, ridogrel, and rosoxacin are superior in their binding stabilities [18]. In another study, we predicted that the drugs lopinavir and valrubicin would have dual-targeting abilities to ACE-2 and TMPRSS2. These drugs could be effectively developed as SARS-CoV-2 entry inhibitors [19]. In the present study, we hypothesized that targeting multiple families of proteins of COVID-19 pathogenesis will have advantages over the current approaches. The molecular docking studies, Induced-Fit Docking, MM-GBSA, followed by MD studies, were performed for the selected class of antiviral, anti-inflammatory, and immunomodulatory drugs, such as chloroquine, hydroxychloroquine, lopinavir, ritonavir, remdesivir, ribavirin, arbidol, favipiravir, darunavir, oseltamivir, azithromycin, tetracycline, teicoplanin, sirolimus, baricitinib, cyclosporine, ivermectin, dexamethasone, nintedanib, resveratrol, quercetin, epigallocatechin 3-gallate betamethasone sodium phosphate, curcumin, andrographolide, nafamostat, camostat, nitazoxanide, and fluvoxamine as ligands by considering the S-protein, ACE-2, and M^pro^ as proteins for all of the studies.

## 2. Materials and Methods

### 2.1. Computational Tools and Study Design

The protein crystal structures were downloaded from the proteins data bank (PDB) and UniProt, while the structures of the ligands were taken from PubChem. The Maestro Molecular modeling platform (Schrödinger, LLC, NY, USA) was used to perform the molecular docking studies, followed by Induced-Fit Docking, Prime MM-GBSA, and MD simulations using Schrodinger software. Molecular docking was used to study the molecular interactions and binding affinity between the selected molecules and the target proteins, which indicates the behavior of the ligands (drugs/molecules) at the target protein site and elucidates the underlying molecular mechanism. Then, Prime MM-GBSA calculations were conducted, followed by Induced-Fit Docking. The top-hit molecules were then subjected to MD simulation to evaluate the binding stability of the ligand on the target protein.

### 2.2. Selection of Suitable Protein Targets

Based on the literature, there are three important protein families that are involved in the pathogenesis of COVID-19, including ACE-2 (PDB ID: 6M1D), M^pro^ (PDB ID: 5R82), and spike protein (PDB ID: 1ZV8). The crystal structure of each protein was selected based on its resolution from PDB (http://www.rcsb.org/ accessed on 11 July 2022).

### 2.3. Preparation of Ligands

In this study, antiviral, anti-inflammatory, and immunomodulatory molecules were selected from the literature based on their mechanism of action, as provided in Table 1. The ligands’ structures were downloaded and incorporated into Maestro. The ligands were then optimized using the LigPrep tool of Schrödinger (LigPrep, Schrödinger, LLC, NY, USA, 2020) to obtain appropriate geometry-optimized stable structures with the lowest energy at neutral pH 7.0 [20].

### 2.4. Protein Preparation

The protein crystal structure was processed using Epik Protein Preparation Wizard (Schrödinger, LLC, NY, USA, 2020), where missing hydrogens, amino acid residues, and side chains were added. A proper ionization state for the protein residues was generated, the water molecules were removed, and the H-bond network was generated. The protein structure was energy minimized using the OPLS3 force field [44]. 

### 2.5. SiteMap

The SiteMap tool was used to identify the druggable ligand-binding pocket in the proteins that lack a bound ligand in their crystal structure to identify the potential binding site for the ligand. The SiteMap module returns potential sites on the protein, which are ranked based on their SiteScore and DScore. Based on these values, a binding site was selected, and the ligands were docked on the proteins [45].

### 2.6. Molecular Docking

The Glide module was used to perform molecular docking. The receptor grid was generated using the receptor-grid-generation tool, which defines the region on the protein onto which the module needs to introduce the ligands for docking. The receptor grid file was loaded, and the prepared ligands were selected from the table. The docking was performed in the extra precision (XP) mode. The docked ligand poses were then analyzed for interaction patterns with the protein; the ligand pose with the best interaction with the protein was selected. The top hits obtained from molecular docking were selected and subjected to induce-fit docking, prime MM-GBSA, and MD simulations [46].

### 2.7. Prime MM-GBSA

MM-GBSA was used to determine the ligand binding energies and ligand strain energies for the top hits in the docking studies. The prime MM-GBSA module of Schrödinger was employed for this purpose. The OPLS3e force field was used with the VSGB solvent model, while the ligands and receptors were taken from the project table and workspace. As the MM-GBSA binding energies are the approximate free energies of binding, a more negative value indicates a stronger binding (reported in kcal/mol).

### 2.8. Induced-Fit Docking

The induced-fit docking (IFD) protocol was carried out using the induced-fit tool in Maestro (Induced-Fit Docking Schrödinger, LLC, NY, USA, 2020) on the selected proteins with the top hits obtained from the docking studies and prime MM-GBSA. The prime reason for performing IFD is to permit flexibility to both ligands and proteins, which is restricted in docking studies. IFD is reported to be robust and accurate in predicting the binding affinity between the ligand and the protein pocket of the protein. The ligand, docking, and protein refinement were carried out using glide and prime, respectively, in the IFD tool.

### 2.9. Molecular Dynamics (MD) Simulation

An MD simulation was performed for the shortlisted molecules from the results of IFD and MM-GBSA using the Desmond module (Desmond Molecular Dynamics System, NY, USA, 2020). Initially, the solvated complex system was prepared using TIP3P as a predefined solvent model, and the iso-osmotic condition was maintained in this stage. The System Builder module was used for the preparation of the above system. The solvated system was minimized using the minimization module. It was then loaded and minimized using the 2000 maximum iterations and 1.0 (kcal/mol/Å) convergence threshold. The minimized solvated system was used to run the MD simulation. The MD simulation was performed with the NPT ensemble for 100 ns at 1.01 bar pressure and a 300K temperature by using the Nose–Hoover chain (1 ns as relaxation time) and Martyna–Tobias–Klier (2.0 ns as relaxation time) as the thermostat and barostat method, respectively. The MD simulation results for all of the ligands were analyzed by generating the simulation interaction diagram [47]. The MD simulation runs were carried out twice to account for possible variations.

## 3. Results

### 3.1. Molecular docking, Prime MM-GBSA and Induced-Fit Docking

Molecular docking was performed on the protein main protease (M^pro^, PDB ID: 5R82) with a known binding site, whereas SiteMap was performed for the proteins in the cases where their crystal structure lacked a bound ligand, due to which the binding site is not clearly known (ACE2 and spike protein, PDB ID: 6M1D and 1ZV8, respectively). SiteMap helps to identify the druggable binding site on the protein based on analysis of the entire protein. The results of the SiteMap analysis were obtained as Site-score and D-score, as depicted in Table 2.

**Figure 1:**
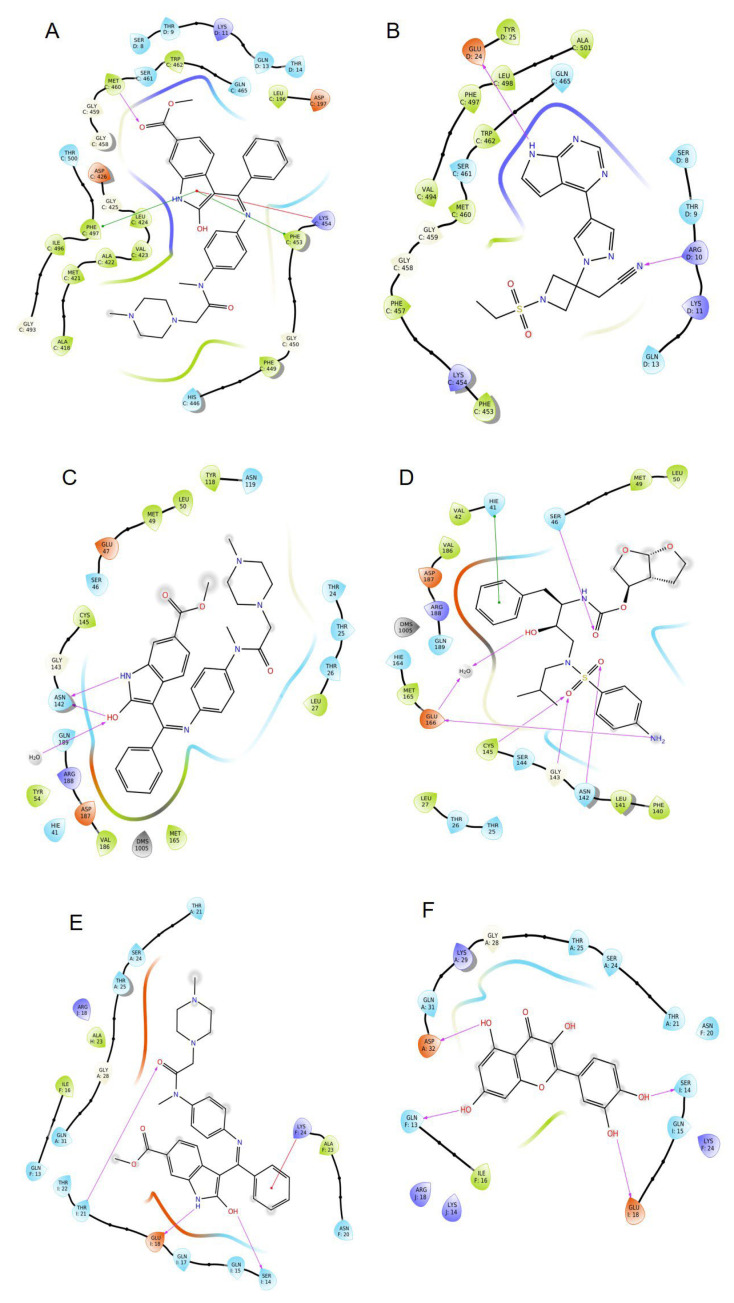
2D ligand-interaction diagram on SARS-CoV-2 proteins. (**A**,**B**): Nintedanib and Baricitinib on ACE2 Protein, PDBID 6M1D; (**C**,**D**): Nintedanib and Darunavir on M^pro^, PDBID 5R82; (**E**,**F**): Nintedanib and Quercetin on Spike Protein. PDBID: 1ZV8.

Based on the SiteMap analysis results for the 1ZV8 protein, site-1 showed the highest Site-Score of 1.022 as well as a D-Score of 1.037 whereas, for the 6M1D protein, site-5 showed the highest Site-Score of 1.11 and D-score of 1.162. Thus, these sites were selected to perform the molecular docking studies. Table 3 summarizes the docking score, IFD score, and ΔG values for the three proteins, while the IFD scores could not be obtained for protein 6M1D. However, the protein docking pose of 6M1D was satisfactory for proceeding with further MD simulation studies. Figure 1 shows the 2D ligand interaction diagrams between the ligands and the proteins. With protein 6M1D site-5 nintedanib, which has the best docking score of −6.056 and a ΔG value of −44.53, while baricitinib followed closely behind with a docking score of −5.76 and ΔG value (−40.66), and ivermectin showed the poorest docking score of −5.328 among the selected ligands and a ΔG value of −17.33. Nintedanib formed a π-cation interaction with LYS454, a π-π stacking interaction with PHE497, and H-bonds with MET460, while baricitinib formed H-bonds with THR9 (Figure 1A,B). In protein 5R82, nintedanib has the best docking score of −5.428 and ΔG value of −50.42, but the IFD score of −643.67 was less when compared with darunavir, which showed a docking score of −4.691, ΔG value of −47.691, and IFD score of −649.42. Fluvoxamine and favipiravir showed docking scores (−4.631 and −3.968) and ΔG values (−42.4 and −23.42), fluvoxamine showed the least IFD score of −641.05, but compared to nintedanib, favipiravir showed a better IFD score (−643.92). Nintedanib formed π-π stacking interactions with HIE41 and H-bonds with SER46, while darunavir showed π-π stacking interactions with HIE41 and H-bonds with ASN142 and GLN189 amino acid residues (Figure 1C,D). Finally, for 1ZV8 site-1, it can be seen that quercetin showed the best docking score of −4.958 and IFD score of −1181.47, but nintedanib showed the best prime MM-GBSA score (ΔG value =−40) while having a docking score of −4.113 and an IFD score of −1172.72, close to that of quercetin. Lopinavir and hydroxychloroquine showed a docking score of −3.521 and −3.464 and a prime MM-GBSA ΔG of −35.61 and −24.44. However, lopinavir has a marginally better IFD score of −1175.01 than that of nintedanib, whereas hydroxychloroquine showed the least IFD score of −1172.02. Nintedanib exhibits amino acid interactions, such as π-cation interactions and H-bonds, with LYS24. In addition to this, it also formed H-bonds with SER14 and GLU18. In comparison, quercetin formed H-bonds with ASP32, THR25, and GLU18 (Figure 1E,F).

### 3.2. Molecular Dynamics (MD) Simulation

**Figure 2:**
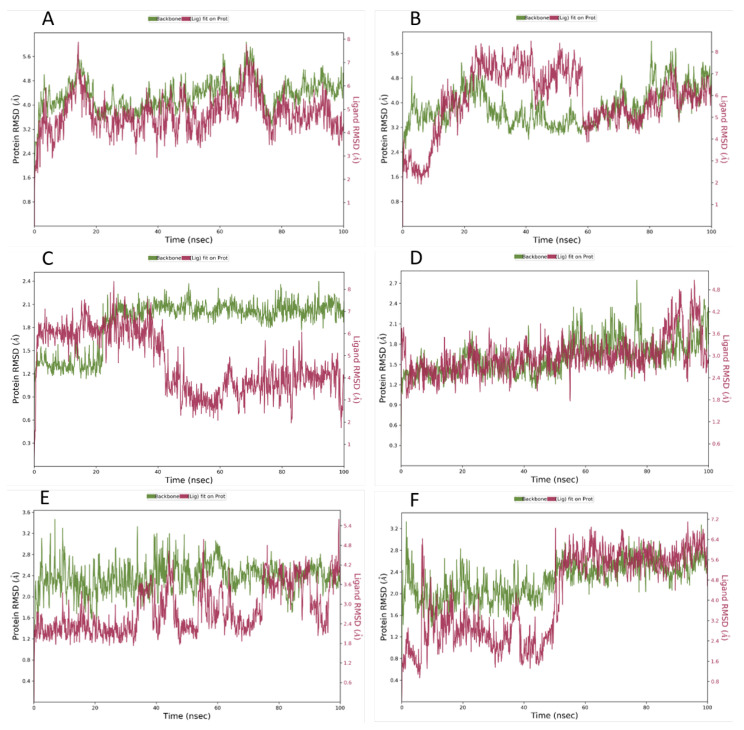
Root mean square deviation (RMSD) plot for ligand–protein interaction. (**A**,**B**): Nintedanib and Baricitinib on ACE2 Protein, PDBID: 6M1D; (**C**,**D**): Nintedanib and Darunavir on M^pro^, PDBID: 5R82; (**E**,**F**): Nintedanib and Quercetin on Spike Protein. PDBID: 1ZV8.

After performing the initial computational studies, the binding mode stability of the selected molecules on the different proteins was analyzed using MD simulation studies. The MD simulation runs were carried out twice to account for possible variation. The simulation interaction diagram shows us various parameters, such as protein root mean square deviations (RMSD), ligand RMSD, protein root mean square fluctuation (RMSF), ligand RMSF, and the interactions observed between the ligand and the amino acids of the protein, among other results. Of all the MD simulation runs, only nintedanib on 6M1D showed a slight deviation between run 1 and run 2, while every other MD simulation run yielded the exact same result. Figure 2A,B represents the P-RMSD and L-RMSD of nintedanib and baricitinib, respectively, on 6M1D. In Figure 2A, the protein 6M1D fluctuation was between 3.2 Å–5.6 Å, while the ligand nintedanib was between 4 Å–7.5 Å for a 100 ns duration. At a given point, the maximum fluctuation was less than 2.5 Å, which indicates the greater stability of the ligand–protein interaction for a longer time. In Figure 2B, the protein (6M1D) fluctuations were between 2.4–5.6 Å, and for ligand baricitinib, the initial fluctuations were between 1.6–5.6 Å at zero to 60 ns, which later stabilized between 3.2–5.6 Å at 60–100 ns. This confirmed that initially, the interactions of the ligand with the protein were less and found to be stable at the end of 60–100 ns. Figure 2C represents the protein (5R82) and ligand nintedanib fluctuations between 1.2–2.1 and 5.5–8 Å, initially at 0–40 ns, while the protein fluctuations decrease to 6.5–7.5 Å and later increase to 2.5–5.5 up to the duration of 100 ns. In Figure 2D, the RMSD fluctuations for protein 5R82 and ligand darunavir were between 1.0–4.2 Å and 2.4–4.8 Å, respectively, which confirmed the formation of stronger and more stable interactions. In Figure 2E, the RMSD fluctuations for protein 1ZV8 and ligand nintedanib were between 1.6–3.2 Å and 1.8–4.2 Å, respectively, which indicates stable interactions between the ligand and the protein. In Figure 2F, the RMSD fluctuations for protein 1ZV8 and ligand quercetin were between 1.6–3.2 Å and 0.8–6.4 Å, respectively, which confirmed stronger and more stable interactions.

**Figure 3:**
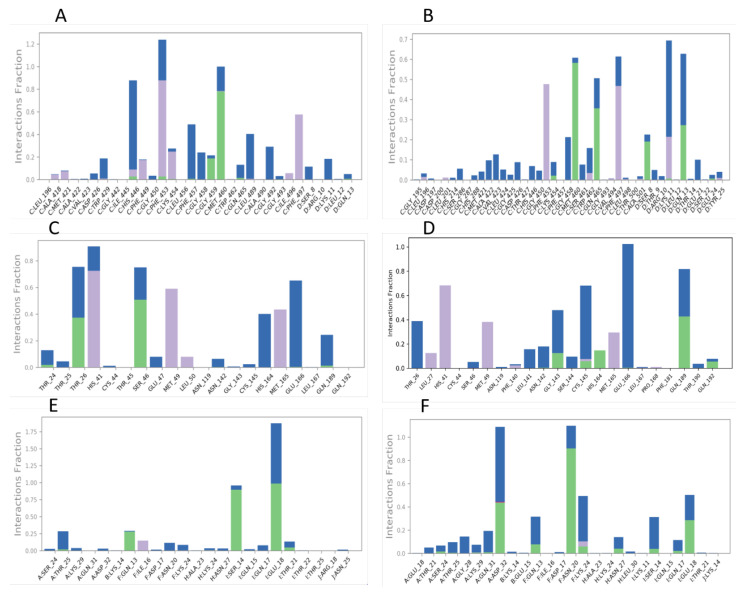
Protein–ligand contacts. (**A**,**B**): Nintedanib and Baricitinib on ACE2 Protein, PDBID: 6M1D; (**C**,**D**): Nintedanib and Darunavir on M^pro^, PDBID: 5R82; (**E**,**F**): Nintedanib and Quercetin on Spike Protein. PDBID: 1ZV8.

In addition to RMSDs, it is also important to know the intermolecular interactions between the ligand and the protein, such as H-bonds, hydrophobic interactions, π-π stacking, π-cation, salt bridges, and water bridges. These can be graphically visualized by protein–ligand contact plots through a bar diagram and are reported as the fraction interaction (in %). Figure 3 represents the protein–ligand contacts seen between the ligands and the proteins. The interactions between nintedanib and baricitinib with 6M1D are represented in Figure 3A,B, respectively, nintedanib forms H-bonds with MET460 for 78% while forming hydrophobic bonds with PHE453 and 497 at 85.3% and 57.6%, respectively, it also forms water bridge interactions with HIS446 at 79%, LEU489 at 39.8%, PHE457 at 48.3% and GLY492 at 29.1%, whereas baricitinib forms H-bonds with MET460 at 58.2%, GLN465 at 35.7%, and GLN13 at 27.3%. Baricitinib forms hydrophobic bonds with PHE453 and 497 at 47.7% and 46.8%, respectively, while showing water bridge interactions with LYS11 at 47.9%. The protein–ligand contacts exhibited in Figure 3C,D are of nintedanib and darunavir on 5R82, respectively. It can be seen that darunavir forms H-bonds at 42.8% with GLN189 while forming hydrophobic bonds with HIS41 at 68.3%, MET49 at 38.2%, and with MET165 at 29.5%. Darunavir forms water bridge interactions with THR26 at 39%, GLY14 at 35.5%, CYS145 at 60.5%, and GL166 at 102%, whereas nintedanib forms H-bonds with THR26 and SER46 at 37.4% and 50.7%, respectively. Hydrophobic bonds are formed with HIS41 at 72.5%, MET49 at 59.1%, and with MET165 at 43.5%, while showing water bridge interactions with HIS164 and GLU166 at 40.2% and 64.9%. Figure 3E,F represent the protein–ligand contacts between nintedanib and quercetin with 1ZV8, respectively; nintedanib forms H-bonds with GLU18 and SER14 at 98.8% and 89.7%, respectively, while forming water bridge interaction with THR25 at 36.7% whereas, quercetin forms H-bonds with ASP32 at 43.8%, ASN20 at 90.4%, and with GLU18 at 28.5%, while forming water bridge interactions with LYS24 and SER14 at 39.7% and 27.4%. Similar to PL contacts, there are ligand–protein (LP) contacts that are mentioned in Table 4.

**Figure 4:**
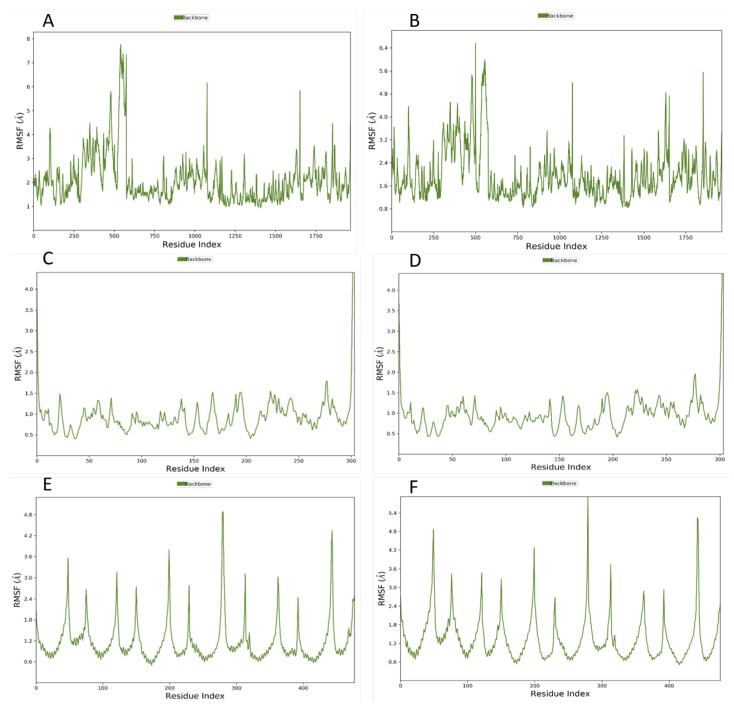
Protein root means square fluctuation (RMSF). (**A**,**B**): Nintedanib and Baricitinib on ACE2 Protein, PDBID: 6M1D; (**C**,**D**): Nintedanib and Darunavir on M^pro^, PDBID: 5R82; (**E**,**F**): Nintedanib and Quercetin Spike Protein. PDBID: 1ZV8.

Additionally, the P-RMSF plot from the simulation interaction diagram (SID) is illustrated in Figure 4. Each peak corresponds to the region of the protein that has fluctuated; usually, the N and C terminals of the protein fluctuate the most. Figure 4A,B represent the P-RMSF when nintedanib and baricitinib are complexed with 6M1D, and it can be seen that the protein fluctuates less when baricitinib is complexed with it, while in the case of 5R82 (Figure 4C,D), the presence of nintedanib and darunavir have rendered the protein equally fluctuated. Figure 4E,F indicate the P-RMSF of 1ZV8 when complexed with nintedanib and quercetin, respectively, and it can be seen that when complexed with nintedanib, the protein is less fluctuated. 

**Figure 5:**
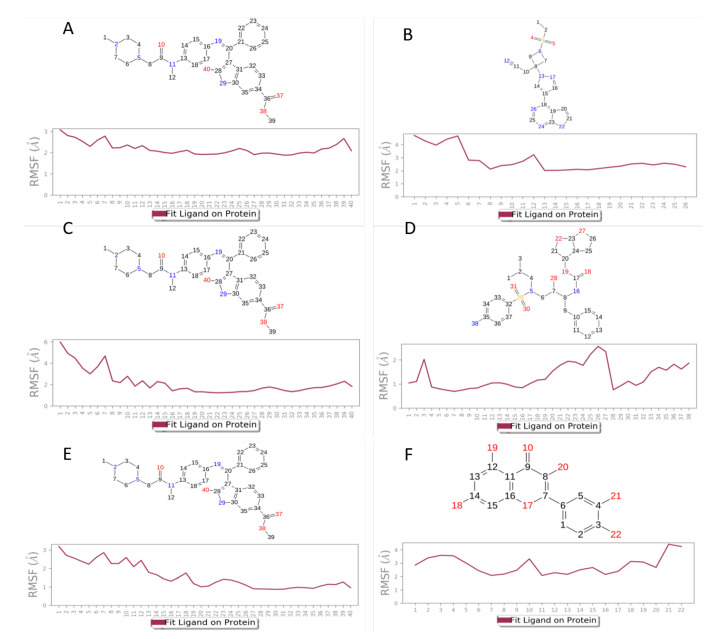
Ligand root means square fluctuation (RMSF). (**A**,**B**): Nintedanib and Baricitinib on ACE2 Protein, PDBID: 6M1D; (**C**,**D**): Nintedanib and Darunavir on M^pro^, PDBID: 5R82; (**E**,**F**): Nintedanib and Quercetin on Spike Protein. PDBID: 1ZV8.

**Figure 6:**
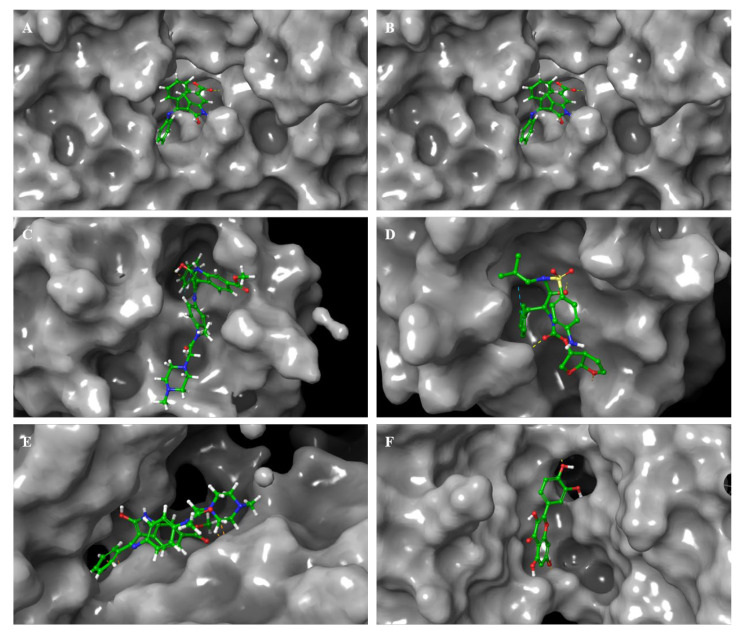
Ligand 3D interactions. (**A**,**B**): Nintedanib and Baricitinib on ACE2 Protein, PDBID: 6M1D; (**C**,**D**): Nintedanib and Darunavir on M^pro^, PDBID: 5R82; (**E**): Nintedanib; (**F**): Quercetin on Spike Protein. PDBID: 1ZV8.

Similar to protein RMSF, Figure 5 illustrates the ligand RMSF and shows the atoms in the ligand that show the highest fluctuation. For nintedanib, the atoms that show the most fluctuations are 10, 37, 38, and 40 across all three proteins. Figure 5B represent the L-RMSF of baricitinib on 6M1D, the atoms that fluctuate the most are atom 3, 4, and 5. The L-RMSF of darunavir on 5R82 is represented by Figure 5D, and it can be seen that atoms 18, 19, 22, 27, 28, 29, 30, and 31 fluctuate the most. The L-RMSF of quercetin on 1ZV8 is shown in Figure 5F; the atoms that fluctuate the most are 10, 17, 18, 19, 20, 21, and 22. Figure 6 represents the 3D conformation of the ligand when it occupies the protein pocket; the dotted lines depict the amino acid interactions. The thermal MMGBSA was performed using the obtained MD simulation trajectory, and the results are discussed in the supplementary data file. The solvent-accessible surface area (SASA) and radius of gyration (RGY) plots (Figures S1 and S2) are obtained through SID and discussed in the supplementary data file.

## 4. Discussion

A considerable number of computational docking and predictions for the treatment of COVID-19 are in progress. The success of repurposed drugs such as remdesivir, favipiravir, and dexamethasone is the stimulus for further computational predictions [48,49]. A study by Zannella et al., 2022, showed that the synthetic three-residue peptide had good interaction with SARS-CoV-2 Spike protein by binding tightly to arginine at position 509 in SARS-CoV-2 RBD (PDB 6XM4) [50]. Our previous study using computational tools demonstrated the SARS-CoV-2 entry inhibitor and shortlisted lopinavir and valrubicin by dual targeting TMPRSS2 and ACE2 [19]. In the current approach, the computationally repurposed drugs are further screened for multiple targets, and the suitable one was proposed for COVID-19 therapy. The ligands selected from the literature survey were initially subjected to molecular docking studies, the ligand which showed the best docking score, along with good amino acid interactions, were then checked for prime MM-GBSA and IFD. The prime MM-GBSA score is represented by ΔG and is indicative of the energy changes involved when a ligand tries to occupy the binding site of a protein. The higher energy spent, the less chance it has to disassociate from that binding site. A greater negative value indicates a higher strength for all three parameters. For ACE-2 protein (6M1D), nintedanib shows the best docking score and ΔG value, followed by baricitinib, the amino acid interactions are also greater in number for nintedanib. The other ligands underperformed and showed poor amino acid interactions with ACE-2 (6M1D), due to which nintedanib and baricitinib were selected for MD simulation. The M^pro^ protein(5R82), nintedanib, had the best docking score and ΔG value, but darunavir had a greater IFD score, fluvoxamine and favipiravir when compared, had decreased docking scores and ΔG than that for nintedanib and darunavir. However, based on the amino acid interactions, it was evident that darunavir has more interactions than nintedanib, while fluvoxamine and favipiravir showed fewer interactions. This was the reason why nintedanib and darunavir were selected for MD simulation, as they outperformed the other ligands. For spike protein (1ZV8), quercetin exhibited the best docking score and IFD score, while nintedanib had a greater ΔG value. Lopinavir and hydroxychloroquine had poorer docking scores among the selected ligands, but lopinavir had an IFD score that was greater than nintedanib and a ΔG value greater than quercetin. Upon inspecting the amino acid interactions, it was clear that nintedanib showed more interactions, followed by quercetin, while lopinavir and hydroxychloroquine had fewer interactions. Hence, quercetin and nintedanib were selected for MD simulation. 

MD simulation studies help to understand the behavior of a docked ligand on the protein in a solvated system over a period of time under simulated biological conditions. For the ACE2 protein, it was seen that nintedanib and baricitinib showed RMSD values within 2.5 Ȧ. The RMSD plots show that nintedanib stays in the protein-binding pocket, which can be understood from Figure 2A. The two lines are pretty overlapping most of the time, whereas it can be seen that the two lines are farther away from each other in Figure 2B, indicating that baricitinib initially separates out from the protein pocket, later sitting in it. When protein–ligand contacts are measured, it can be seen that nintedanib has a greater number of interactions crossing the 50% mark with amino acids (Figure 3A,B) than what is shown by baricitinib. This increased number of interactions with the protein might be the reason why nintedanib stays in the protein-binding pocket but baricitinib does not. Although the P-RMSF (Figure 4A,B) and L-RMSF (Figure 5A,B) seem slightly better for baricitinib, they have fewer protein fluctuations when baricitinib is complexed; it is not superior to nintedanib as the difference is not much. Based on these results, it is clear that for ACE-2, nintedanib shows better affinity amongst all of the ligands. In the case of the M^pro^ protein, from the RMSD plot (Figure 2D), it is clear that darunavir stays in the protein-binding pocket for the entire simulation period while nintedanib does not (Figure 2C). The RMSD value is greater than 2.5 Ȧ for nintedanib but not for darunavir. A similar trend is being seen in the case of protein-ligand contacts (Figure 3C,D); darunavir has a greater number of interactions with amino acids when compared with nintedanib. P-RMSF (Figure 4C,D) is the same for both proteins, indicating that the ligand has fluctuated the proteins equally, while L-RMSF (Figure 5C,D) is better for darunavir. From the results, it is understood that for M^pro^, darunavir shows better affinity, while nintedanib follows closely behind. Finally, for spike protein (1ZV8), nintedanib and quercetin show an RMSD value within 2.5 Ȧ, and both ligands do not occupy the protein-binding pocket for the entire duration of the simulation (Figure 2E,F). The amino acid interaction between the ligand and protein was seen to be equal for both nintedanib and quercetin (Figure 3E,F), but nintedanib showed interactions of better quality and stability. The P-RMSF plot reveals that when complexed with nintedanib, the protein seems to be less fluctuated when compared with quercetin (Figure 4E,F) while L-RMSF (Figure 5E,F) shows that nintedanib is less fluctuated when compared with quercetin. From there, it can be stated that nintedanib shows a better affinity for the spike protein. From the MD results, it can be concluded that across all three proteins, nintedanib can be selected as a ligand for multi-targeting as it shows superior affinity with all three proteins when compared with the rest of the proteins.

Currently, COVID-19 management options are supportive therapy and preventive control measures in hospital settings. It takes a great deal of time for USFDA to receive and approve drugs specific to SARS-CoV-2, due to which researchers resort to repurposing existing approved molecules for the management of COVID-19. Drugs such as favipiravir, nelfinavir, ivermectin, lopinavir, remdesivir, baricitinib, and tocilizumab are being tested clinically to evaluate their effectiveness against SARS-CoV-2 [7]. Though the drug remdesivir is approved for use against COVID-19, there are limitations to its effective use in most patients [51]. The route of administration of remdesivir is also a concern for the effective management of COVID-19 [52]. All of these attempts are focused on a single family of protein in SARS-CoV-2, but in actuality, more than one prominent family of proteins plays a major role in the functioning of SARS-CoV-2. In this drug repurposing study, we focused on three major families of proteins and tried to identify one drug molecule that can bind to all three proteins, thereby enabling multi-targeting. A single molecule has the ability to interact with the three proteins, which will result in better efficacy. Of the molecules selected, nintedanib, quercetin, darunavir, and baricitinib showed the most promising interactions with the three proteins across molecular docking, induced-fit docking, prime MM-GBSA, and molecular dynamic simulations. The molecular docking score (−4.113), ∆G value (−40), and IFD (−1172.72) score of nintedanib were superior to the remaining selected drugs, which was closely followed by quercetin for 1ZV8 protein. In the case of the M^pro^ protein, nintedanib has the highest docking score (−5.482) and ∆G (−50.42), but the IFD score (−643.67) was less when compared with darunavir. For ACE-2, nintedanib showed the highest docking score (−6.056) and ∆G value (−44.53). In the MD simulation of nintedanib, after 40 ns of simulation separated away from the M^pro^, respectively, protein-binding pocket, whereas it was stable with the spike protein and ACE-2. 

Nintedanib is used in the treatment of idiopathic pulmonary fibrosis (IPF), systemic sclerosis-associated interstitial lung disease, and non-small cell lung cancer along with docetaxel [53]. Nintedanib is a kinase inhibitor that has an affinity for viral proteins and shows its action on pulmonary proteins as well. Patients with post-COVID-19 pulmonary fibrosis and COVID-19 pneumonia have responded positively to nintedanib treatment. It has been found to reduce the incidence of the acute exacerbation of IPF in COVID-19 patients. It was reported that the repurposing of nintedanib has been beneficial for treating COVID-19 patients to treat severe lung fibrosis. Clinical trials (phase-II) are ongoing with nintedanib to check the safety and efficacy of treating idiopathic pulmonary fibrosis in COVID-19 patients [54]. The pathophysiological similarities, such as inflammation and difficulty in breathing, which exists between IPF and COVID-19, suggest that the pathogenic mechanism that leads to pulmonary fibrosis in these two conditions are the same, hence nintedanib can be used to manage COVID-19. 

## 5. Conclusions

Based on the results of the molecular docking score, ΔG value, and the IFD score, the best molecules, nintedanib, quercetin, darunavir, and baricitinib, were shortlisted. The most preferred interaction was seen in nintedanib in the MD simulation with spike proteins, M^pro^, and ACE2 proteins. Thus, nintedanib could be a suitable molecule for the treatment of COVID-19, but in vitro and in vivo experimental results should substantiate these findings.

## Figures and Tables

**Table 1:** List of ligands shortlisted for computational studies.

Drug Name	Category of Drug	Mechanism of Action on SARS-CoV-2	References
Arbidol	Antiviral	Interacts with viral lipid envelope and clathrin-mediated endocytosis	[21]
Azithromycin	Antibiotic	Increases endosomal pH and inhibits the replication of the virus	[22]
Andrographolide	Antiviral and anti-inflammatory	Inhibits SARS-CoV-2 M^pro^/3CLpro	[23]
Baricitinib	Immunosuppressant	Inhibits Janus kinase	[24]
Betamethasone sodium phosphate	Corticosteroid, immunomodulatory	Inhibits the synthesis of inflammatory mediators	[25]
Camostat	Serine protease inhibitor in pancreatitis	Inhibits TMPRSS2 protein	[26]
Chloroquine	Anti-malarial	Increasing endosomal pH	[27]
Curcumin	Polyphenol (anti-viral), anti-inflammatory	Prevents entry of the virus into host cells and inhibits SARS-Co-V-2 protease	[28]
Cyclosporine	Immunosuppressant	Inhibits RdRp	[29]
Darunavir	Antiviral	By protease inhibition	[30]
Dexamethasone	Corticosteroid, immunomodulatory	Alters inflammatory agents and immune responses	[31]
Epigallocatechin-3 gallate	Flavonoid, anti-inflammatory	Inhibits SARS-CoV-2 M^pro^/3CLpro	[32]
Favipiravir	Antiviral	Inhibits RdRp	[33]
Fluvoxamine	Anti-depressant	Inhibits viral M^pro^/3CLpro	[34]
Hydroxy chloroquine	Anti-malarial	Interaction with ACE-2 and glycosylation	[35]
Ivermectin	Anthelmintic	Inhibits the replication of the virus	[36]
Lopinavir and ritonavir	Antiviral	Inhibits M^pro^/3CLpro	[37]
Nafamostat	Serine protease inhibitor in pancreatitis	Inhibits TMPRSS2 protein	[38]
Nintedanib	Anti-fibrotic agent	Inhibits tyrosine kinases	[39]
Nitazoxanide	Anti-protozoal and anti-viral	Inhibits the production of pro-inflammatory cytokines	[22]
Oseltamivir	Antiviral	Inhibits neuraminidase	[24]
Quercetin	Flavonoid, anti-inflammatory	Inhibits SARS-CoV-2 M^pro^/3CLpro	[40]
Remdesivir	Antiviral	Inhibits the replication of the virus	[24]
Resveratrol	Anti-viral, anti-inflammatory (Polyphenol)	Inhibits both the replication of the virus and the synthesis of proteins	[40]
Ribavirin	Antiviral	Prevents synthesis of mRNA-capping polymerase and replication of virus	[24]
Sirolimus	Immunosuppressant	Inhibits the mTOR signaling pathway	[41]
Teicoplanin	Antibiotic	Targets spike protein at the cleavage site on cathepsin L	[42]
Tetracycline	Antibiotic	Forms complexes with the zinc present in the viral cellular components and decreases cytokines	[43]

**Table 2:** Site-score and D-score for the sites on 1ZV8 and 6M1D.

Sites	Proteins			
	1ZV8		6M1D	
	Site-Score	D-Score	Site-Score	D-Score
Site-1	1.022	1.037	1.045	0.971
Site-2	0.920	0.932	1.032	1.026
Site-3	0.878	0.876	1.038	1.056
Site-4	0.978	0.929	1.107	1.043
Site-5	1.014	1.004	1.110	1.162

**Table 3:** Docking score, MM-GBSA and IFD-Score.

Protein PDB ID	Ligands	Docking Score	MM-GBSA	IFD-Score
6M1D	Nintedanib	−6.056	−44.32	-
	Baricitinib	−5.76	−40.66	-
	Ivermectin	−5.328	−17.33	-
5R82	Nintedanib	−5.482	−50.42	−643.67
	Darunavir	−4.691	−47.28	−649.44
	Fluvoxamine	−4.631	−42.4	−641.05
	Favipiravir	−3.698	−23.42	−643.92
1ZV8	Quercetin	−4.985	−35.45	−1181.47
	Nintedanib	−4.113	−40	−1172.72
	Lopinavir	−3.521	−35.61	−1175.01
	Hydroxychloroquine	−3.464	−24.44	−1172.02

**Table 4:** Ligand-protein contacts exhibited during MD simulation.

Protein	Ligand	L-P Contacts %
M^pro^—5R82	Darunavir	34 (THR26)
		68 (HIS41)
		32 (GLY143)
		57 (CYS145)
		77 (GLU166)
		42 (GLN189)
	Nintedanib	72 (HIS41)
		31 (SER46)
		35 (HIS164)
		32 (GLU166)
ACE2—6M1D	Baricitinib	31 (LYS11)
		58 (MET460)
		33 (GLN465)
	Nintedanib	35 (HIS446)
		35 (PHE453)
		44 (PHE457)
		78 (MET460)
		39 (LEU489)
Soike Protein—1ZV8	Nintedanib	89 (SER14)
		98 (GLU18)
	Quercetin	46 (ASN20)
		38 (ASP32)

## Data Availability

The generated data are used in the manuscript, and some data are provided as supplementary files.

## Supplementary Materials

Supplementary file is provided with this manuscript. Further, the following supporting information can be downloaded at: https://www.researchsquare.com/article/rs-101359/v1 (accessed on 1 October 2022).
